# The relationship between the deep fibular nerve and the dorsalis pedis artery and its surgical importance

**DOI:** 10.4103/0970-0358.53007

**Published:** 2009

**Authors:** R. Chitra

**Affiliations:** Assistant Professor, Department of Anatomy, Siddhartha Medical College, Vijayawada, Andhra Pradesh, India

**Keywords:** Anterior tarsal tunnel, Deep fibular nerve, Dorsalis pedis artery, Dorsum of foot

## Abstract

The aim of this study was to demonstrate the relationship between the deep fibular nerve and the dorsalis pedis artery to provide useful anatomical knowledge for safe surgical approaches in plastic surgery. The dissection of 30 cadaver lower limbs was undertaken to describe the relationship of the deep fibular nerve to the dorsalis pedis artery in the anterior tarsal tunnel and on the dorsum of the foot. The anterior tarsal tunnel is a flattened space between the inferior extensor retinaculum and the fascia overlying the talus and navicular. The deep fibular nerve and its branches pass longitudinally through this fibro-osseous tunnel, deep to the tendons of the extensor hallucis longus and extensor digitorum longus. Four distinct relationships of the deep fibular nerve to the dorsalis pedis artery were determined. The dorsalis pedis neurovascular island flap contains both the dorsalis pedis artery and the deep fibular nerve. Because the design of a neurovascular free flap requires detailed knowledge of the nerve and vascular supply, the data presented here are intended to help surgeons during surgical approaches to the foot and ankle.

## INTRODUCTION

The deep fibular nerve descends with the anterior tibial artery to the ankle, dividing there into lateral and medial terminal branches. The deep fibular nerve is related laterally to the dorsalis pedis artery at the ankle. The medial terminal branch runs distally on the dorsum of the foot lateral to the dorsalis pedis artery and connects with the medial branch of the superficial peroneal nerve in the first interosseous space. It divides into two dorsal digital nerves, which supply adjacent sides of the great and second toes. Before dividing, it gives off an interosseous branch that supplies the first metatarsophalangeal joint and the first dorsal interosseous muscle. The deep fibular nerve may end as three terminal branches.[1] The lateral terminal branch crosses the ankle deep to the extensor digitorum brevis, enlarges as a pseudoganglion, and supplies the extensor digitorum brevis. From the enlargement, the branches of the nerve supply the tarsal and metatarsophalangeal joints of the 2^nd^, 3^rd^, and 4^th^ toes. The first branch also supplies the second dorsal interosseous muscle.[1]

The dorsalis pedis neurovascular island flap contains both the dorsalis pedis artery and the deep fibular nerve.[2,3] The aim of this study was to demonstrate the relationship between the deep fibular nerve and the dorsalis pedis artery to provide useful anatomical knowledge for safe surgical approaches in plastic surgery.

## MATERIAL AND METHODS

During routine educational dissections conducted by undergraduate students of our department of anatomy for 3 consecutive academic years (2005-2006 batch, 2006-2007 batch, and 2007-2008 batch), the relationship between the deep fibular nerve and the dorsalis pedis artery were observed in both lower limbs of 10 cadavers (7 males and 3 females) and in 10 disarticulated lower limbs (Right-5, Left-5) where gender was unknown.

After skin removal, the deep fascia of the leg and the dorsal fascia of the foot were incised parallel to the cutaneous incision. The inferior extensor retinaculum was removed and deep fibular nerve alongwith anterior tibial vessels was identified within the anterior tarsal tunnel. The deep fibular nerve and the artery with their terminal branches were dissected carefully in a proximal to distal direction and the relationship between them was observed and recorded.

## RESULTS

Four distinct relationships of the deep fibular nerve to the dorsalis pedis artery were determined as described by the study conducted by Zuhre,*et al*.[4]

### Type 1

**Figure 1:**
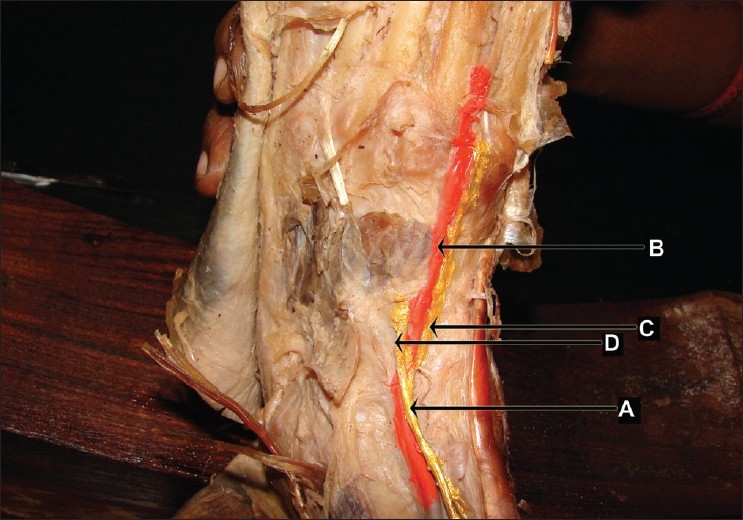
Medial branch of deep fibular nerve medial to dorsalis pedis artery (left lower limb- artery painted red colour and nerves painted yellow colour) (A) deep fibular nerve, (B) dorsalis pedis artery, (C) medial branch of deep fibular nerve, (D) lateral branch of deep fibular nerve

The dorsalis pedis artery was medial to the deep fibular nerve in the tunnel and medial to the medial terminal branch distal to the tunnel on the dorsum of the foot [Figure 1]. This pattern was observed in 36.7% of the limbs (11/30 limbs) in the present study.

### Type 2

**Figure 2:**
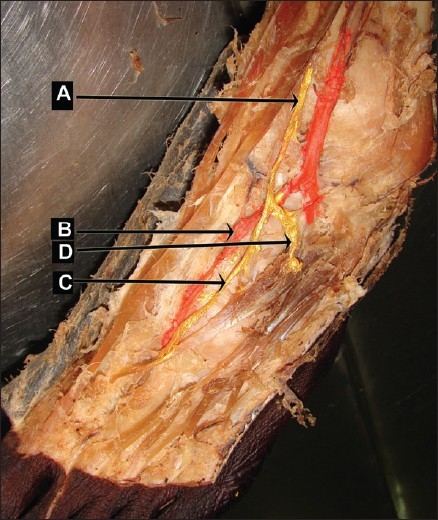
Medial branch of deep fibular nerve lateral to dorsalis pedis artery (Left lower limb - artery painted red colour and nerves painted yellow colour) (A) deep fibular nerve, (b) dorsalis pedis artery, (c) medial branch of deep fibular nerve (d) lateral branch of deep fibular nerve

The artery was medial to the deep fibular nerve in the tunnel and lateral to the medial terminal branch distal to the tunnel on the dorsum of the foot [Figure 2]. This pattern was observed in 30% of the limbs (9/30 limbs) in the present study.

### Type 3

**Figure 3:**
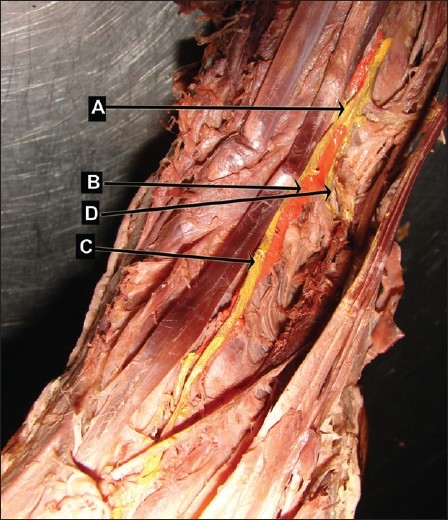
Deep fibular nerve and dorsalis pedis artery crossing at various sites (left lower limb artery painted red, nerve painted yellow) (A) deep fibular nerve, (B) dorsalis pedis artery, (C) medial branch of deep fibular nerve, (D) lateral branch of deep fibular nerve

The deep fibular nerve and the artery crossed over each other at multiple levels. This pattern was observed in 26.7% of the limbs (8/30 limbs) in the present study [Figure 3].

### Type 4

**Figure 4:**
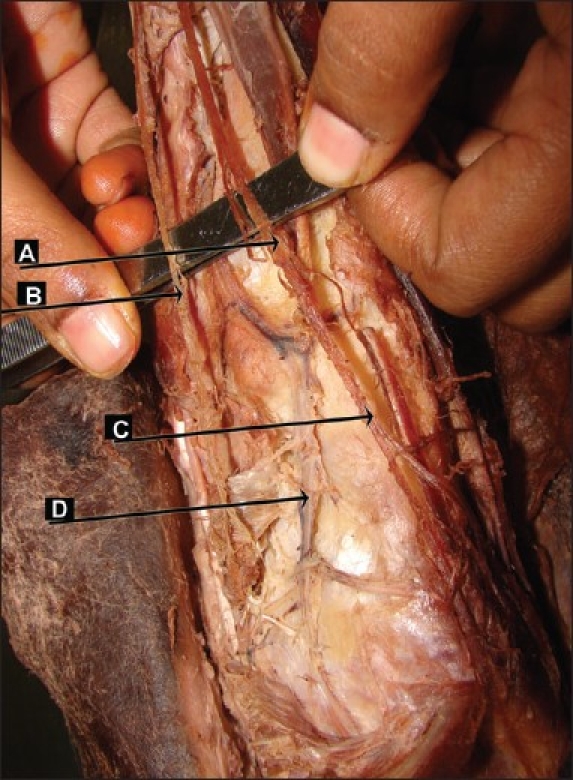
Absence of medial branch of deep fibular nerve (right lower limb) (A) deep fibular nerve, (B) superficial fibular nerve, (C) lateral branch of deep fibular nerve, (D) dorsalis pedis artery

No medial terminal branch of the deep fibular nerve was observed. The artery was medial to the lateral terminal branch. This pattern was observed in 6.7% of the limbs (2/30 limbs) in the present study [Figure 4].

In the present study, when a type was observed in a lower limb, the other side tended to fall into some other type in 70% of the cases. In the case of Type 4, the absence of the medial branch was noted in two limbs (1 right and 1 left) whereas medial branch was found medial to the dorsalis pedis artery on the contralateral limb in these bodies. In the case of Type 3 in 8 limbs (5 left and 3 right), other patterns such as Types 1 and 2 were observed in the other sided limbs except in the case of the bilateral Type 3 pattern.

## DISCUSSION

The dorsalis pedis flap is being widely used for reconstruction in many cases including eye socket, intraoral, palm, and hand reconstructions.[5–7] Because this flap contains the dorsalis pedis artery and deep fibular nerve, both anatomical structures have great importance in flap surgery.[5–9] The congenital absence of the dorsalis pedis artery was noted at the rate of 2.25% by Demetrios Chavatzas[10] using an ultrasonic technique. The extensor digitorum brevis has been variously used to treat facial paralysis. Advances in microsurgery have provided new methods for using the extensor digitorum brevis, namely, transferring the muscle with neurovascular anastomoses. The arterial supply of this muscle is the anterior tibial artery through the lateral tarsal branches of the dorsalis pedis artery. The deep fibular nerve provides motor innervation to the extensor digitorum brevis by means of the lateral tarsal branches.[7,11] The extensor digitorum brevis was supplied by the accessory peroneal nerve, a branch of the superficial peroneal nerve in 25% of the cases in the study conducted by Neundorfer, *et al*.[12] Thus, additional clinical significance of the dorsalis pedis artery and the deep fibular nerve is attributable to their relationship with the extensor digitorum brevis. The anatomy of these structures is very relevant in the dissection of first or second toes for toe-to-hand transfer, dissection of the extensor digitorum brevis muscle flap as a local or free flap, etc.

Rab, *et al*.[13] studied 28 feet from 14 cadavers to investigate the deep fibular nerve. Their results contradicted with the results of the present study in which the dorsalis pedis artery was positioned posteromedially at the inferior extensor retinaculum in 26 out of 28 specimens and anteromedially in 2 of 28 specimens. Rab, *et al*. did not observe any crossing between the deep fibular nerve and the artery. Our results are similar to the study conducted by Zuhre, *et al.*[4] in which four different patterns of relationship between the deep fibular nerve and the dorsalis pedis artery were observed.

We consider that when the artery crosses over the nerve in Type 3, there is a risk of entrapment of the deep fibular nerve by the dorsalis pedis artery aneurysms.[14] Anatomical knowledge will be helpful to foot and ankle surgeons during the surgical release of the nerve. Because the design of a neurovascular free dorsalis pedis flap requires detailed knowledge of the nerve and vascular supply, the data presented here is intended to help surgeons during surgical approaches to the foot and ankle.
